# Formulation and Evaluation of Once Daily Minocycline Hydrochloride Extended Release Matrix Tablets

**DOI:** 10.4103/0250-474X.56034

**Published:** 2009

**Authors:** R. V. Keny, S. A. Mankame, C. F. Lourenco

**Affiliations:** Department of Pharmaceutics, Goa College of Pharmacy, Panaji, Goa-403 001, India

**Keywords:** Minocycline hydrochloride, hydroxypropylmethylcellulose, ethyl cellulose, extended release, matrix tablets

## Abstract

The present study was aimed to develop once daily extended release matrix tablets of minocycline hydrochloride, using hydroxypropylmethylcellulose either alone or in combination with ethyl cellulose as the matrix material in different proportions. The formulated tablets were also compared with a marketed product. The results of the dissolution study indicate that formulations FC-IV, FC-V and FC-VI showed maximum drug release upto 24 h, whereas the marketed product was found to extend the release only up to 14 h. Incase of formulations containing combination of hydroxypropylmethylcellulose and ethyl cellulose (FC-I to FC-IX), the release of the drug was found to be dependent on the relative proportions of hydroxypropylmethylcellulose and ethyl cellulose used in the tablet matrix. Mathematical treatment of the *in vitro* drug release data suggests that, all the formulations best fitted into first order release kinetics. Drug release from the matrix occurred by combination of two mechanisms, diffusion of drug from tablet matrix and erosion of tablet surface, which was reflected from Higuchi's model and Erosion plot.

Minocycline hydrochloride (minocycline HCl) is a semi synthetic tetracycline, which has been primarily indicated for the treatment of *Acne vulgaris*, where its success has been attributed to a combination of its bacteriostatic and antiinflammatory activities[1]. But its use is limited due to acute vestibular adverse events (AVAEs). In order to lower overall systemic exposure to reduce unwanted side effects, a study was initiated to develop an extended release dosage form of minocycline[2]. It has a half life of 10 to 12 h and the usual oral dosage for treatment of *Acne vulgaris* has been 50-100 mg twice daily[3]. The drug is freely soluble in water[4] and hence judicious selection of release retarding excipients is necessary to achieve a constant *in vivo* input rate of the drug[5]. The matrix tablets composed of drug and the release retarding material (polymer), offers the simplest approach in designing an extended-release system[6]. Number of studies shows the use of hydrophilic matrices to formulate the controlled release dosage forms of different drugs[7–11]. Because of their simplicity and cost-effectiveness, hydrophilic gel matrix tablets are widely used for oral controlled release dosage forms. Hydrophilic polymers form a gel like structure around the tablet core which controls the drug release. The hydrophilic polymer hydroxypropylmethylcellulose (HPMC), selected in the present study is a pH-independent material which has been widely used to prepare extended release dosage forms such as promethazine and acetaminophen[12]. However the use of hydrophilic polymer alone for controlling the drug release of highly water soluble drugs is restricted due to rapid diffusion of the dissolved drug through the hydrophilic gel layer. Use of hydrophobic polymers will retard the drug release of such drugs with high water solubility. Thus hydrophobic polymers are suitable, along with a hydrophilic matrix for developing extended-release dosage forms[5]. Since ethylcellulose (EC) is a hydrophobic polymer and cannot swell in a manner similar to HPMC, it was considered that admixture of HPMC with EC could change the permeability of the matrix and consequently modify the drug release rate[13].

Hence, in the present work, an attempt has been made to develop extended-release matrix tablets of minocycline using putative hydrophilic matrix materials such as HPMC, alone and in combination with the EC as the hydrophobic polymer[14–16], and to study the *in vitro* release characteristics and kinetics of the prepared formulations. The kinetics of the dissolution process was studied by the application of five kinetic equations to the dissolution data namely, the zero-order, the first-order, the Higuchi-square root, Korsmeyer-Peppas equation and erosion plot. The prepared formulations were also compared with a marketed product (MP) (Solodyn90, Medics Pharma) which contained HPMC 2910 and carnauba wax as the matrix material.

## MATERIALS AND METHODS

Minocycline HCl USP was a gift sample from Wyeth Laboratories (Goa, India). HPMC (HPMC K4M and K15M) and EC (20 cps) were obtained as gift samples from Colorcon Asia Pvt Ltd. (Goa, India). Lactose IP and polyvinylpyrrolidone (PVP-K30) were gifted by Loba Chemie (Mumbai, India). Materials and excipients used in preparing tablets were of IP grade. All other ingredients used throughout the study were of analytical grade and were used as received.

### Preparation of matrix tablets:

Different tablet formulations (Batch size of 50 tablets) were prepared by wet granulation technique i.e. F-I to F-VI (Table 1) and FC-I to FC-IX (Table 2). Accurately weighed quantities of pre-sieved drug, lactose and matrix material (HPMC and EC) were mixed uniformly, and wetted with 10% w/v solution of PVP in IPA as granulating fluid (13 ml for F-I to F-VI and 9 ml incase of FC-I to FC-IX), the cohesive mass thus obtained was screened through a sieve No. 12. The granules were air dried at room temperature in an enclosure to protect it from light. The coarse granules so obtained were once again screened using the same sieve. Talc and magnesium stearate were finally added as anti-frictional agents to the uniformly sized granules and the granules were compressed (10 mm diameter, biconvex punches) using a single punch tablet compression machine (Cadmach, Ahmedabad). Each tablet contained 90 mg of minocycline hydrochloride and other excipients as listed in Tables 1 and 2. Prior to compression, the granules were evaluated for various IPQC tests.

**TABLE 1:** TABLET FORMULATIONS WITH SINGLE MATRIX MATERIAL

Ingredients (mg/tablet)	F-I	F-II	F-III	F-IV	F-V	F-VI
Minocycline HCl	90	90	90	90	90	90
HPMC K4M	70	105	140	-	-	-
HPMC K15M	-	-	-	70	105	140
Lactose	153.5	118.5	83.5	153.5	118.5	83.5
PVP-K30*	26	26	26	26	26	26
Talc	7	7	7	7	7	7
Magnesium stearate	3.5	3.5	3.5	3.5	3.5	3.5

Defined bulk weight per tablet is 350 mg containing 90 mg of minocycline HCl. F-I to F-VI represents the various formulations of minocycline HCl. Formulation F-I was matrix tablet prepared using 20% HPMC K4M; formulation F-II was matrix tablet prepared using 30% HPMC K4M; formulation F-III was matrix tablet prepared using 40% HPMC K4M; formulation F-IV was matrix tablet prepared using 20% HPMC K15M; formulation F-V was matrix tablet prepared using 30% HPMC K15M and formulation F-VI was matrix tablet prepared using 40% HPMC K15M

* PVP-K30 was used as a 10% solution in IPA.

**TABLE 2:** TABLET FORMULATIONS WITH COMBINED MATRIX MATERIAL OF HPMC K15M AND EC

Ingredients (mg/tablet)	Set-I			Set-II			Set-III		
			
	FC-I 2:1	FC-II 3:1	FC-III 4:1	FC-IV 2:1	FC-V 3:1	FC-VI 4:1	FC-VII 2:1	FC-VIII 3:1	FC-IX 4:1
Minocycline HCl	90	90	90	90	90	90	90	90	90
HPMC K15M	70	78.75	84	93.35	105	112	116.66	131.25	140
EC	35	26.25	21	46.66	35	28	58.35	43.75	35
Lactose	126.5	126.5	126.5	91.5	91.5	91.5	56.5	56.5	56.5
PVP-K30*	18	18	18	18	18	18	18	18	18
Talc	7	7	7	7	7	7	7	7	7
Magnesium stearate	3.5	3.5	3.5	3.5	3.5	3.5	3.5	3.5	3.5

Defined bulk weight per tablet is 350 mg containing 90 mg of minocycline HCl. Set-I contain 30% w/w of the combined matrix, set-II contains 40% w/w of the combined matrix and set-III contains 50% w/w of the combined matrix. FC-I to FC-IX represents the various formulations of minocycline HCl. Ratios indicate HPMC K15: EC proportions.

* PVP-K30 was used as a 10% solution in IPA.

### Evaluation of Granules:

Both loose bulk density (LBD) and tapped bulk density (TBD) were determined. A quantity of 2 g of granules from each formulation, previously lightly shaken to break any agglomerates, was introduced into a 10 ml measuring cylinder. After the initial volume was observed, the cylinder was allowed to fall under its own weight onto a hard surface from the height of 2.5 cm at 2 sec intervals. After 300 taps, the tapped volume of packing was noted. LBD and TBD were calculated using the formulae[17]; LBD= weight of the powder/volume of the packing, TBD= weight of the powder/tapped volume of the packing.

The compressibility index of the granules was determined by Carr's compressibility index[18]; Carr's index (%) = [(TBD-LBD)×100]/TBD. The angle of repose of granules was determined by the funnel method. The accurately weighed granules were taken in a funnel, which was maintained at 4 inches from the surface. The granules were allowed to flow through the funnel freely onto the surface. The diameter of the powder cone was measured and angle of repose was calculated using the equation[19]; tan θ = h/r, θ = tan^−1^(h/r), where θ is the angle of repose, h is height in cm of the powder cone and r the radius in cm of the powder cone. Moisture content of granules was determined using Karl Fischer instrument (Spectralab, Mumbai). The granules were weighed and added into the reagent solution of the instrument, which was stirred and the tare weight was fed into the instrument. Then after certain duration of time the moisture content as %w/w was read on the monitor.

### Evaluation of matrix tablets:

The prepared matrix tablets were evaluated for hardness, weight variation, thickness, diameter, friability and drug content. Tablet hardness was determined for 10 tablets using a Monsanto hardness tester (Campbell Electronics, Mumbai). The weight variation was evaluated on 20 tablets using an electronic balance (Precisa 310M, Switzerland), and the test was performed according to the official method[20]. The thickness and diameter was determined for 10 tablets with the help of a digital Vernier calliper (Mitutoyo, Japan). Friability was determined taking 20 tablets in a Roche friabilator (Electrolab, Mumbai) for 4 min at 25 rpm. Drug content of the matrix tablets was determined by weighing and finely grinding 10 tablets of each batch. Aliquot of this powder equivalent to 90 mg of minocycline HCl was accurately weighed, suspended in approximately 50 ml of phosphate buffer pH 7.2 and shaken for 15 min. Final volume was adjusted to 100 ml with phosphate buffer and filtered. From this 10 ml was diluted to 100 ml. The final volume was made by taking 2 ml of above solution and diluted to 10 ml with phosphate buffer. Absorbance of this solution was recorded at 245.4 nm using UV/Vis spectrophotometer (Perkin Elmer, Mumbai) against a reagent blank and the content was compared from a calibration curve prepared with standard minocycline HCl in the same medium.

### *In vitro* release rate studies:

**Fig. 1:**
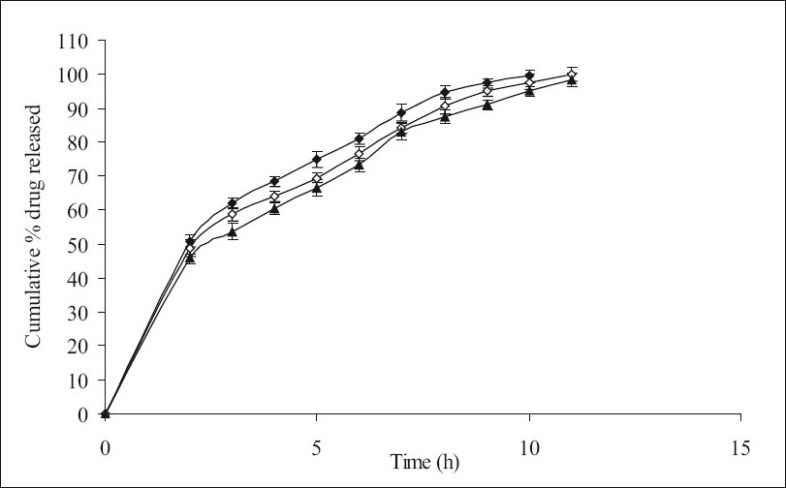
The *in vitro* release profiles of minocycline from F-I, F-II and F-III. Release profiles of minocycline from formulations containing varying HPMC K4M content; F-I [HPMC K4M, 20%] (-♦-), F-II [HPMC K4M, 30%] (-◊-) and F-III [HPMC K4M, 40%] (-▲-). Each data point represents mean±SD (*n* = 6).

**Fig. 2:**
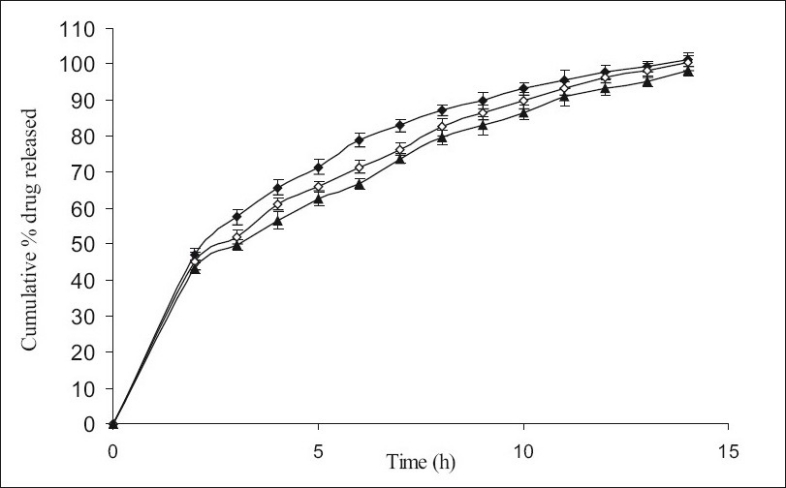
The *in vitro* release profiles of minocycline from F-IV, F-V and F-VI. Release profiles of minocycline from formulations containing varying HPMC K15M content; F-IV [HPMC K15M, 20%] (-♦-), F-V [HPMC K15M, 30%] (-◊-) and F-VI [HPMC K15M, 40%] (-▲-). Each data point represents mean±SD (*n* = 6).

**Fig. 3:**
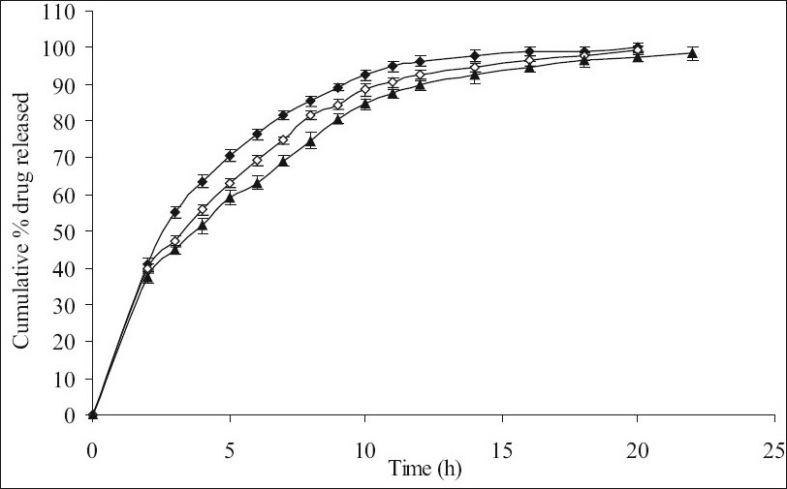
The *in vitro* release profiles of minocycline from FC-I, FC-II and FC-III. Release profiles of minocycline from formulations containing varying HPMC K15M:EC content; FC-I [HPMC K15M, 20%; EC, 10%] (-♦-), FC-II [HPMC K15M, 22.5%; EC, 7.5%] (-◊-) and FC III [HPMC K15M, 24%; EC, 6%] (-▲-). All formulations contain 30% of combined matrix. Each data point represents mean±SD (*n* = 6).

**Fig. 4:**
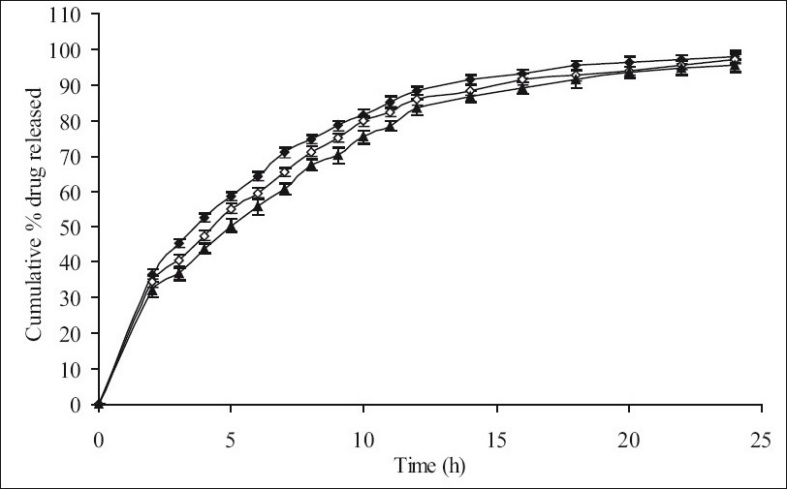
The *in vitro* release profiles of minocycline from FC-IV, FC-V and FC-VI. Release profiles of minocycline from formulations containing varying HPMC K15M:EC content; FC-IV [HPMC K15M, 26.67%; EC, 13.33%] (-♦-), FC-V [HPMC K15M, 30%; EC, 10%] (-◊-) and FCVI [HPMC K15M, 32%; EC, 8%] (-▲-). All formulations contain 40% of combined matrix. Each data point represents mean±SD (*n* = 6).

**Fig. 5:**
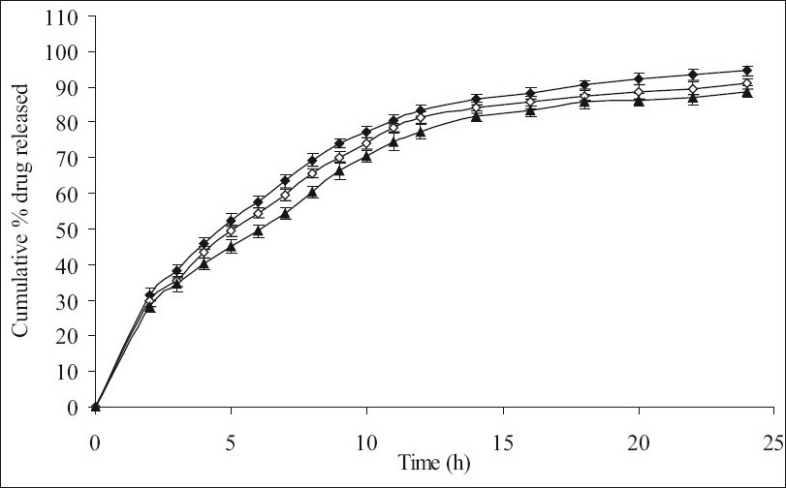
The *in vitro* release profiles of minocycline from FC-VII, FCVIII and FC-IX. Release profiles of minocycline from formulations containing varying HPMC K15M:EC content; FC-VII [HPMC K15M, 33.33%; EC, 16.67%] (-♦-), FC-VIII [HPMC K15M, 37.50%; EC, 12.50%] (-◊-) and FC-IX [HPMC K15M, 40%; EC, 10%] (-▲-) formulations. All formulations contain 50% of combined matrix. Each data point represents mean±SD (*n* = 6).

**Fig. 6:**
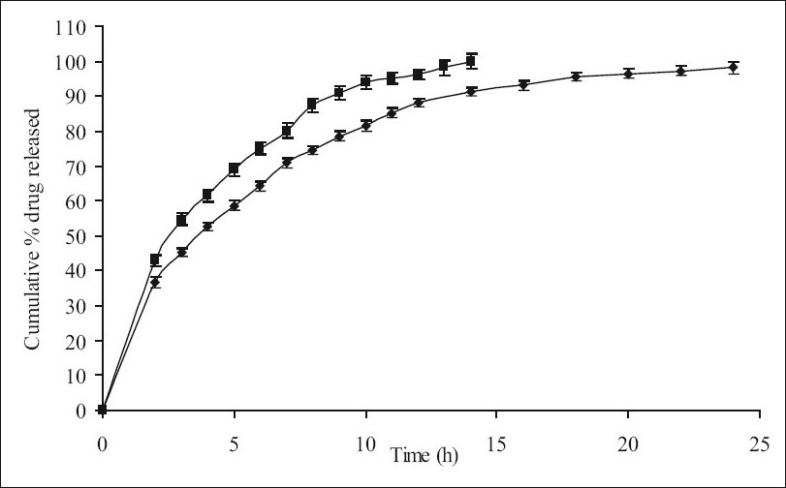
The *in vitro* release profiles of minocycline from FC-IV and Marketed Product. Release profiles of minocycline from formulations; FC-IV [HPMC K15M, 26.67%; EC, 13.33%] (-♦-) and Marketed Product [MP] (-▪-). Marketed product (-▪-) used was Solodyn90, Medics Pharma, USA. Each data point represents mean±SD (*n* = 6).

The *in vitro* release rate studies were carried out in USP dissolution test apparatus Type I (Labindia, Mumbai) in simulated gastric fluid (pH 1.2±0.1) from 0 to 2 h and simulated intestinal fluid (pH 7.2±0.1) from 2 to 24 h. Rotation speed of 50 rpm at temperature of 37±0.5° and dissolution medium of 900 ml was maintained throughout the experiment[8]. At predetermined time intervals, 10 ml of sample was withdrawn and replaced with the same volume pre-warmed (37±0.5°) fresh dissolution medium. The samples withdrawn were filtered through 0.45 μ membrane filters, and drug content in each sample was analyzed after suitable dilution by UV/Vis spectrophotometer at two different wavelengths, 265 nm for samples in gastric fluid and 245.4 nm for samples in intestinal fluid. The actual content in samples was read from a calibration curve prepared with standard minocycline HCl. All dissolution studies were carried out in duplicate and repeated at least thrice. The same was carried out on marketed product for comparative evaluation. The results are shown in figs. 1 to 6.

## RESULTS AND DISCUSSION

Formulation of granules is the key factor in the production of tablet dosage form involving extended release of drug from matrix type particle. Physical parameters such as area, hardness, surface characteristics and size can significantly affect the rate of dissolution of drugs contained in a complex system. The selection of wet granulation technique for matrix tablet preparation was based on previously reported study which suggested that wet granulation results in harder tablets with lower matrix porosity that give very low release rates when compared to direct compression[21]. In our study 10% PVP in IPA was used as granulating agent. Non-aqueous granulating fluid was used, since it was thought to avoid the use of water and heat during drying of granules.

The granules of different formulations were evaluated for LBD, TBD, compressibility index, angle of repose and moisture content. The LBD and TBD of granules ranged from 0.22±0.06 to 0.36±0.03 g/ml and 0.27±0.01 to 0.37±0.06 g/ml respectively. The LBD and TBD of granules F-I to F-VI were found to be lower than formulations of FC-I to FC-IX, which may be attributed to absence of EC in formulations F-I to F-VI. Granules prepared with HPMC alone showed compressibility index values ranging from 8.92±2.14 to 11.81±3.57%, while granules prepared with combination of HPMC and EC, showed values from 9.33±1.78 to 14.55±0.89%. Generally, compressibility index values upto 15% result in good to excellent flow properties, but readings above 25% indicates poor flowability[18]. Angle of repose values of all formulations ranged from 25.96±0.13° to 36.77±0.37°. Generally values of angle of repose are rarely less than 20°, and values upto 40° indicate reasonable flow properties[22]. All these results indicate that the formulated granules possessed satisfactory flow properties and compressibility[17–19,23]. The moisture content of all the formulations was found to be satisfactory.

The results of hardness and friability of the prepared matrix tablets ranged from 6.0±0.35 to 10.0±0.21 kg/cm^2^ and 0.27±0.03% to 0.40±0.07%, respectively. The tablet formulations in all the prepared batches contained minocycline HCl ranging from 97.76±1.12% to 103.22±0.74%. As such, all the batches of the fabricated tablets were of good quality with regard to hardness, friability and drug content. The results of thickness and diameter of tablets ranged from 3.38±0.07 to 3.85±0.03 mm and 10.0±0.03 to 10.1±0.01 mm, respectively. Thus all formulations showed uniform thickness and diameter. Weight variation results of the matrix tablets ranged from 348±1.19 to 353±1.90 mg. For weight variation test, the pharmacopoeial limit for the percentage deviation for tablets of more than 250 mg is ±5%. The average percentage deviation of all tablet formulations was found to be within the above limit, in compliance with official standards[20].

Fig. 1 shows the effect of different concentrations of HPMC K4M 20% (F-I), 30% (F-II) and 40% wt/wt of tablet (F-III) on % release (99.34, 97.71 and 95.03% within 11 h of dissolution study, respectively) of minocycline HCl. No significant difference in release rate was observed between tablets containing either 20 or 30 or 40% of HPMC K4M. However, 40-50% of drug was released in first 2 h of dissolution. Similarly Fig. 2 shows the effect of different concentrations of HPMC K15M 20% (F-IV), 30% (F-V) and 40% wt/wt of tablet (F-VI) on % release (101.36, 100.36 and 98.15 within 14 h of dissolution study, respectively) of the drug. However 40-50% of drug was released in the first 2 h of dissolution study. The % release of formulations with HPMC K15M (F-IV to F-VI) was found to be extended than that of formulations with HPMC K4M (F-I to F-III) due to the use of a more viscous grade of HPMC.

The release of drug depends not only on the nature of matrix but also upon the drug polymer ratio. As the percentage of polymer increased, the kinetics of release decreased. This may be due to structural reorganization of hydrophilic HPMC polymer. Increase in concentration of HPMC may result in increase in the tortuosity or gel strength of the polymer. When HPMC polymer is exposed to aqueous medium, it undergoes rapid hydration and chain relaxation to form viscose gelatinous layer (gel layer). Failure to generate a uniform and coherent gel may cause rapid drug release[24].

Therefore, in the next batch of tablets, to control the release of the drug further, ethyl cellulose was included in the matrix tablets in combination with HPMC K15M and three sets (containing different concentrations of the combined matrix material of HPMC K15M and EC) were prepared. Each set contained 3 different formulations with 3 different ratios (2:1, 3:1 and 4:1) of HPMC and EC. Initially 30% w/w of combined matrix proportion was selected to prepare set-I formulations, so as to keep the amount of matrix to minimum. These formulations showed a release of about 100.07% (FC-I), 99.37% (FC-II) and 97.49% (FC-III) of the drug at the end of 20 h, respectively (fig. 3) with 35-42% drug released at the end of first 2 h. In this set of formulations it seemed that the drug was insufficiently surrounded with the hydrophobic component resulting in burst effect on the drug release in the initial stages of dissolution especially in FC-I resulting in total drug release at the end 20 h. Thus to overcome this, matrix proportion was increased to 40% w/w in set-II formulations. Its cumulative% drug release reached up to 98.13 (FC-IV), 97.22 (FC-V) and 95.49 (FC-VI), respectively at the end of 24 h with less than 40% of drug being released after first 2 h (fig. 4). Set-III formulations showed a release of 94.03% (FC-VII), 90.87% (FC-VIII) and 88.62% (FC-IX) at the end of 24 h which was lesser than formulations of set-II in 24 h and with only upto 34% drug released in first 2 h (fig. 5).

The results indicate slow extended release of the drug in the dissolution media with an increase in HPMC:EC matrix proportion and being complimented by retarding effect of EC on drug release. The drug release profiles were characterized by an initial burst effect with a proportionately greater amount of the drug release, followed by a more sustained release of the drug. The increase in HPMC content with FC-I to FC-IX exhibited controlled rate of drug release respectively, and further as the time progresses swelling of HPMC occurs resulting in extending the duration of drug release. The presence of hydrophobic EC provides a complimentary environment in controlling the drug release from the matrix. However the increase in concentration of EC does not seem to influence the release profile which is predominantly influenced by the increased HPMC content. Nevertheless, the combination effect of HPMC and EC slows down the diffusion process which is found to be relatively uniform from 8 to 24 h. Comparison of the cumulative percent drug release with respect to formulations of set-I, set-II and set-III, it is observed as anticipated that the further increase in HPMC content with proportionate presence of EC sustains the rate of drug release extending the duration quite significantly; since it has been previously reported that EC reduce drug release rates on account of formation of a strong matrix with reduced porosity. This increases diffusional path leading to reduced water penetration through the micropores resulting in slower drug release[21]; For example, in set-II the % drug release between 95 to 98% and set-III between 88 to 94% at the end of 24 h. Comparing the cumulative% drug release of all formulations, FC-IV (40% of combined matrix with 2:1 ratio of HPMC K15M and EC) showed the best release (98.13%) in 24 h, whereas the MP showed a release of 99.96% in 14 h only (fig. 6).

In order to describe the kinetics of the release process of drug in all the formulations as well as in the marketed preparation, various equations were used, such as the zero-order rate equation, which describes the systems where the release rate is independent of the concentration of the dissolved species[25]. The first-order equation describes the release from systems where dissolution rate is dependent on the concentration of the dissolving species[26]. The Higuchi square root equation describes the release from systems where the solid drug is dispersed in an insoluble matrix, and the rate of drug release is related to the rate of drug diffusion[27]. The Korsmeyer-Peppas equation is used to analyze the release of pharmaceutical polymeric dosage forms, when the release mechanism is not well known or when more than one type of release phenomena could be involved[28]. The erosion equation describes the drug release from slabs, spheres and infinite cylinders displaying heterogeneous erosion[29]. The applicability of all of these equations was tested in this work. The kinetics data for all the models is shown in Table 3.

**TABLE 3:** KINETIC VALUES OBTAINED FROM DIFFERENT PLOTS OF FORMULATIONS, F-I TO F-VI, FC-I TO FC-IX AND MP

Formulations	Zero order plots	First order plots	Higuchi's plots	Korsmeyer -Peppas plot		Erosion plot
					
	r^a^	r^a^	r^a^	n^b^	r^a^	r^a^
F-I	0.9385	0.9768	0.9927	0.4124	0.9922	0.9766
F-II	0.8988	0.9835	0.9836	0.4411	0.9907	0.9973
F-III	0.9167	0.9531	0.9811	0.4705	0.9839	0.9913
F-IV	0.9669	0.9977	0.9748	0.4469	0.9868	0.9857
F-V	0.9266	0.9982	0.9916	0.4239	0.9968	0.9977
F-VI	0.9328	0.9941	0.9953	0.4417	0.9963	0.9988
FC-I	0.9324	0.9955	0.9916	0.3690	0.9754	0.9913
FC-II	0.8951	0.9873	0.9979	0.4133	0.9575	0.9899
FC-III	0.9464	0.9897	0.9851	0.4243	0.9644	0.9907
FC-IV	0.9267	0.9941	0.9941	0.3996	0.9857	0.9952
FC-V	0.9588	0.9908	0.9937	0.4358	0.9926	0.9831
FC-VI	0.8827	0.9887	0.9868	0.4417	0.9719	0.9843
FC-VII	0.9227	0.9985	0.9936	0.4547	0.9827	0.9898
FC-VIII	0.8827	0.9952	0.9927	0.4683	0.9951	0.9958
FC-IX	0.9085	0.9976	0.9979	0.4893	0.9855	0.9938
MP	0.9612	0.9922	0.9892	0.4344	0.9808	0.9912

F-I to FC-IX represents the various formulations of minocycline HCl and MP is the Marketed Product.

a Correlation coefficient.

b The diffusional exponent is based on Korsmeyer-Peppas equation, M_t_ /M_∞_= kt^n^.

It is evident from Table 3 that the drug release process is not zero-order in nature. This indicates that the dissolution rate of the drug is independent of the amount of drug available for dissolution and diffusion from the matrix. The dissolution data of all formulations when fitted in accordance with the first-order equation it is evident that a linear relationship was obtained with ‘r’ (correlation coefficient) value close to unity and higher than ‘r’ obtained from the zero-order equation for all formulations, showing that the release is an apparent first-order process. This indicates that the amount of drug released is dependent on the matrix drug load. As concentration reduces on drug release, the diffusional path increases resulting in drug release at a comparatively slower rate in the later phase, thus fitting into Higuchi's kinetics[27]. All the formulations in this investigation could be best expressed by Higuchi's classical diffusion equation, as the plots showed high linearity (r: 0.9748 to 0.9979). The linearity of the plots indicates that the release process is diffusion-controlled. To confirm the diffusion mechanism, the data were fit into Korsmeyer-Peppas model[28]. All the formulations showed good linearity (r: 0.9575 to 0.9968), with slope (n) values ranging from 0.3690 to 0.4893. The ‘n’ values were less than 0.5 indicating drug release by Fickian diffusion. Further the data were plotted for the release by Erosion mechanism[29]. Linearity of the plots (r: 0.9766 to 0.9988) indicates the release mechanism is also by erosion of the matrix. However, drug release from MP shows a zero order release profile initially upto 9 h followed by first order kinetics upto 14 h exhibiting mixed order kinetics. The ‘r’ values indicate compliance with Higuchi's and Erosion models and ‘n’ values confirms that the MP obeys Fick's law.

From these studies it reflected that the presence of HPMC as one of the components in formulations FC-I to FC-IX, acts as a wicking agent, which provides a complimentary environment for hydrophobic EC in controlling the drug release from the matrix. As regards to formulations F-I to F-VI, the drug release depends mainly on the drug diffusion rate through hydrated layer of the macromolecule HPMC. Overall it can be concluded that, the percent of total matrix material as well as the presence of ethyl cellulose significantly influenced the release rate of drug. The tablets with 40% of matrix material (2:1 ratio of HPMC K15M and EC) gave satisfactory results on the formulation of extended release dosage form of minocycline HCl. Thus, above findings suggests that, proportion of HPMC and combinations of HPMC: EC matrices can be optimized to design a once daily preparation of minocycline HCl with drug release being extended upto 24 h.
